# Biofortification of Cereals and Pulses Using New Breeding Techniques: Current and Future Perspectives

**DOI:** 10.3389/fnut.2021.721728

**Published:** 2021-10-07

**Authors:** Rahil Shahzad, Shakra Jamil, Shakeel Ahmad, Amina Nisar, Sipper Khan, Zarmaha Amina, Shamsa Kanwal, Hafiz Muhammad Usman Aslam, Rafaqat Ali Gill, Weijun Zhou

**Affiliations:** ^1^Agricultural Biotechnology Research Institute, Ayub Agricultural Research Institute, Faisalabad, Pakistan; ^2^Maize Research Station, Ayub Agricultural Research Institute, Faisalabad, Pakistan; ^3^Department of Plant Breeding and Genetics, University of Agriculture, Faisalabad, Pakistan; ^4^Tropics and Subtropics Group, Institute of Agricultural Engineering, University of Hohenheim, Stuttgart, Germany; ^5^Institute of Plant Protection, Muhammad Nawaz Sharif University of Agriculture, Multan, Pakistan; ^6^Key Laboratory of Biology and Genetic Improvement of Oil Crops, The Ministry of Agriculture and Rural Affairs, Oil Crops Research Institute of Chinese Academy of Agricultural Sciences, Wuhan, China; ^7^Key Laboratory of Spectroscopy Sensing, The Ministry of Agriculture and Rural Affairs, Institute of Crop Science, Zhejiang University, Hangzhou, China

**Keywords:** anti-nutrients, CRISPR-Cas, conventional breeding, fertilization, malnutrition, micronutrients, genome editing, transgenic breeding

## Abstract

Cereals and pulses are consumed as a staple food in low-income countries for the fulfillment of daily dietary requirements and as a source of micronutrients. However, they are failing to offer balanced nutrition due to deficiencies of some essential compounds, macronutrients, and micronutrients, i.e., cereals are deficient in iron, zinc, some essential amino acids, and quality proteins. Meanwhile, the pulses are rich in anti-nutrient compounds that restrict the bioavailability of micronutrients. As a result, the population is suffering from malnutrition and resultantly different diseases, i.e., anemia, beriberi, pellagra, night blindness, rickets, and scurvy are common in the society. These facts highlight the need for the biofortification of cereals and pulses for the provision of balanced diets to masses and reduction of malnutrition. Biofortification of crops may be achieved through conventional approaches or new breeding techniques (NBTs). Conventional approaches for biofortification cover mineral fertilization through foliar or soil application, microbe-mediated enhanced uptake of nutrients, and conventional crossing of plants to obtain the desired combination of genes for balanced nutrient uptake and bioavailability. Whereas, NBTs rely on gene silencing, gene editing, overexpression, and gene transfer from other species for the acquisition of balanced nutritional profiles in mutant plants. Thus, we have highlighted the significance of conventional and NBTs for the biofortification of cereals and pulses. Current and future perspectives and opportunities are also discussed. Further, the regulatory aspects of newly developed biofortified transgenic and/or non-transgenic crop varieties *via* NBTs are also presented.

## Introduction

The products of cereal and pulses (Box 1) are used as a staple food in developing and developed countries and serve as a source of nutrients and dietary energy. On average, cereals are composed of ~75% carbohydrates (mainly starch), 6–15% proteins in full-grain, which may vary from species to species, and contribute ~50% of the global energy terms. The importance of cereals can be judged from the fact that global food security to a greater degree depends on their availability which amounts to 2,600 million tons annually (1). The changing climate is putting heavy pressure on crop production with the increasing demand for crops that can withstand harsh climatic conditions, i.e., drought and heat stress, and can deliver a balanced diet to human beings (2, 3). Under these circumstances, pulses have emerged as an important component of the food chain, which can provide an environmentally stable source of protein, fats, and micronutrients (Box 1). Pulses are great sources of complex carbohydrates, dietary proteins, minerals, and vitamins for human nutrition. These are excessively used in various parts of the world as traditional diets because of high protein concentration, balanced amino acid profiling, and slow digestibility of carbohydrates. These are popularly used because of the delivery of proteins and micronutrients and balanced diets to the masses in a cost-efficient manner. These along with cereals offer a complete diet if biofortified for nutrient compounds (4, 5).

Box 1 **Terms and definitions related to biofortification of crops.**

**Cereals:** A cereal is any grass cultivated for edible components of its grain, which is composed of germ, endosperm, and bran.

**Pulses:** Pulses are a type of leguminous crop that are harvested solely for the dry seed. Dried beans, lentils, and peas are the most known and consumed types of pulses. Pulses do not include crops that are harvested green (e.g., green peas, green beans).

**Biofortification:** It is a process of increasing the nutritive value of a food crop through use of fertilizer, selective breeding, or genetic modification.

**Macronutrient:** Macronutrients could be defined as chemical elements or a class of chemical compounds that are consumed in large quantities by the human body for the sake of energy for growth, metabolism, and other body functions.

**Micronutrient:** Micronutrients include dietary minerals and vitamins that are required in very small quantities (<100 mg per day) and not involved in regulation of growth directly. However, these are vital for health development, disease prevention, and well-being.

**Malnutrition:** Malnutrition is a condition that results from eating a diet which does not supply a healthy amount of one or more nutrient. These include diets that either contain too much nutrient or not enough nutrient.

**Over-nutrition:** It is a form of malnutrition that arises due to intake of a diet having insufficient energy and nutrient amount.

**Hidden hunger:** It describes a state of deficiency of essential vitamins and minerals in the human diet.

**Food fortification:** It is the process of adding micronutrients to the food with the aim of delivering a balanced diet.

**Dietary diversification:** It is a process of changing the dietary preferences of the household, i.e., increasing the uptake of animal-sourced food.

**Supplementation:** It is a term used to describe the provision or relatively large amount of micronutrients in the form of pills, tablets, capsules, or syrups to improve nutrition health in the short term.

**Agronomic biofortification:** It describe the biofortification method in which deliberate application of mineral fertilization is carried out to increase the concentration of the desired micronutrients in the edible part of the crop to increase dietary intake.

**Conventional breeding:** It is a process of development of new varieties of crops by using older techniques and natural processes without using the latest molecular plant biological tools.

**New breeding techniques (NBTs):** These are crop improvement techniques that make specific changes with the plant DNA in order to change the trait of interest. These modifications may vary from single base pair addition, deletion, substitution to removal, or addition of complete gene in an organism.

**Transgenic breeding:** It refers to the genetic improvement of crop plants in relation to various economic traits useful for human beings by means of genetic engineering tools.

**Genetic engineering:** It is a process of using the recombinant DNA technology to alter the genetic make-up of an organism.

**RNA interference:** This term is used to describe a cellular mechanism that uses a gene's own DNA sequence to turn it off in a process called gene silencing. In plants, animals, and fungi, RNAi is triggered by double-stranded RNA (dsRNA).

**Genome editing:** It is a crop improvement technique in which DNA is inserted, deleted, modified, or replaced in the genome at a particular location without disturbing the rest of the genome.

**Transgenic crops:** Transgenic or genetically modified crops are plants that have DNA modified using genetic engineering methods.

**Biofortified crops:** These are described as nutritionally enhanced food crops offering increased bioavailability of different nutrients to the human population.

**Overexpression:** It is a process of making too many copies of a plant protein by attaching an upstream constitutive promotor using genetic engineering tools.

**Mega-nucleases:** These are characterized by a large recognition site (double-stranded DNA sequences of 12–40 base pairs) which gives them an advantage of having only a unique target site once in a genome to achieve target specificity. These are equally effective for modification of genomes of all organisms.

**Zinc finger nucleases (ZFNs):** These are artificially designed nucleases which are comprised of zinc finger DNA binding domain attached to a DNA cleavage domain. Zinc finger domain can be engineered to target specific target DNA sequences; resultantly, ZFNs target a specific gene in a complex genome of large organisms to achieve target specificity.

**Transcription activator-like effector nuclease (TALENs):** These are restrictions enzymes that can be engineered to cut DNA at a specific target site. These are also comprised of two domains, i.e., TAL effector binding DNA domain and DNA cleavage domain.

**Clustered regularly interspaced short palindromic repeats (CRISPR)/Cas9:** It is the simplest, most versatile, and precise method of genome editing which is comprised of two components, i.e., Cas9 enzyme which acts as a pair of molecular scissors which can cut the two strands of DNA at a specific location in the genome which can be added or removed and guide-RNA (gRNA) which is a small piece of predesigned RNA sequence (about 20 bases long) located inside a longer RNA scaffold. Upon binding of the scaffold to the target DNA, gRNA guide Cas9 and make sure that the cleavage takes place at the target site.

**Base editing:** It is a CRISPR based gene editing system which offers introduction of point mutations at the desired target site without generating double stranded breaks. Two major classes of base editors have been identified, i.e., cytidine base editors (CBE) which allow C>T conversion and adenine base editors (ABE) which allow A>G conversions.

**Prime editing (PE):** It is a search and replace genome editing tool in which pegRNA complex binds to the target DNA and Cas9 nicks only one strand generating a flap and allowing donner free precise genome editing. PE not only allows all sorts of transitions and transversions, but also allows small insertions and deletions.

**Anti-nutrients compounds:** These are natural or synthetic compounds that interfere with the adsorption of essential nutrients and make them unavailable to human beings, e.g., phytate, lectins, tannins, protease inhibitors, and calcium oxalate.

The United Nations (UNs) has set 17 sustainable development goals (SDGs) in 2015. Out of which, SDG3 is about “ensuring healthy lives and promote well-being for all at all ages.” Good health starts with nutrition; however, without regular and nutritious food humans cannot live, learn, fend off diseases, or lead productive lives (https://sdgs.un.org/goals/goal3). According to the European Food Safety Authority report, the daily dietary reference values of several nutrient elements for adults are 8–11 mg for zinc, 8–18 mg for iron, and 750 mg for calcium, depending on gender, which is not met in our daily diets, leading to micronutrient deficiency (6). Micronutrient deficiency is a silent epidemic—it slowly weakens the immune system, stunting physical and intellectual growth, and even causing death (7). Micronutrient deficiency, also known as hidden hunger (Box 1), is extremely pronounced with more than 2 billion masses affected by it (8). This deficiency escalates the probability of infectious diseases and deaths resulting from diarrhea, measles, malaria, and pneumonia in numerous low-income countries. Food fortification (Box 1) through supplementation and crop biofortification is considered as an industrialized solution for this alarming nutritional deficiency (9). Biofortification (Box 1) is a micronutrient-enriching approach, involving strategies focused on targeting and modulation of movement pathways, i.e., root uptake phenomenon, transporting, remobilizing, storage, and increased bioavailability of the minerals. The four main strategies widely employed in crops' biofortification include agronomic biofortification focused on better mineral solubilizing and mobilization while conventional plant breeding, genetic engineering, and gene editing mainly focus on cultivar improvement in terms of micronutrient accumulation, bioavailability, and suppression of anti-nutrients (Box 1) (10).

Biofortification was initially designed to overcome the drawbacks and deficiencies of supplementation (11). Therefore, a modern weapon against mineral deficiency involved transferring the genes directly in elite genotypes. Transgenic crops employed the genetic engineering tools for genotype improvement in focused metabolic pathways of the plant to improve and modify carbohydrates, fats, proteins, minerals, vitamins, and other secondary metabolites (12). These transgenes targeted redistribution of micronutrients between tissues, enhanced the efficiency of a biochemical pathway, increased bioavailability, reduced anti-nutrient absorption and multigene transfer (corn with a high concentration of beta-carotene, ascorbate, and folate in a multivitamin plant) with the reconstruction of selected pathways (field of system biology) (12). With the advent of various “Omics” technologies, different CRISPR-based gene-editing tools including CRISPR-Cas9/13 (Box 1) and transcription activator-like effector nucleases (TALENs) (Box 1) as well as availability of sequence genome of multiple species opened new horizons for crop biofortification (13, 14). Because of the above-mentioned facts, we have exploited the potential of new breeding techniques (NBTs), i.e., genetic engineering, RNAi, and gene editing for the development of nutritionally enriched crops to combat malnutrition (Box 1). Further regulatory aspects of crops developed through NBT and prospects were also discussed in light of the latest developments.

## Malnutrition—A Hidden Hunger

Malnutrition is an emerging threat in an ever-increasing population around the globe. The world population is expected to rise to 8.3 billion in 2030 against the present-day 7.8 billion. According to UNFAO, 792.5 million people around the globe suffer from malnutrition with more victims in the developing world. Most people in developing countries either face hunger or receive nutrient-poor food. The global death rate due to hunger piles up to 24,000 people per day (15). About one-third of the population is suffering from “hidden hunger.” They lack either one or all of the essential micro- or macronutrients (Box 1), like Zn, Fe, selenium, iodine, folic acid, lysine, vitamin A, vitamin B12, vitamin C, vitamin D, and others (Table 1), in their diets (12, 33). These vitamins and nutrients are inevitable for the growth, development, and proper functioning of the human body. Deficiency of these micronutrients for a short period does not cause much harm but their shortage for a long time may cause many diseases like anemia (Fe deficiency), beriberi (Vitamin B deficiency), pellagra (niacin deficiency), rickets (vitamin D deficiency), and scurvy (vitamin C deficiency) or be fatal (Table 1) (34, 35). Approximately 25% of the world's population suffers from anemia and Fe deficiency is its leading cause (50–60% affected). Beriberi is responsible for 50% of the deaths of children in the early 1920s in rice-consuming regions around the globe (36). Rickets is a very serious threat to many communities around the globe, i.e., China has 3%, Bangladesh 13%, Magnolia 25.6%, India 13%, and the Middle East 10% population affected by this disease (37). These facts highlight the importance of biofortification of crops to deliver healthy diets to the world's population to overcome these issues.

**Table 1:** Essential micro- and macro-nutrients required for good human health.

**Nutrient**	**Composition in cereals**	**Quantity in pulses**	**Deficiency symptoms in humans**	**References**
**Micronutrients (mg/100 g food)**				
Iron (Fe)	0.500–3.800	1.250–3.330	Anemia	(16, 17)
Zinc (Zn)	0.600–3.300	1.060–1.530	Wilson's disease, sickle cell disease, chronic kidney disease, chronic liver disease	(16, 17)
Copper (Cu)	0.001–0.012	0.180–0.290	Anemia, paleness, neurological disorders, muscle weakness	(18, 19)
Manganese (Mn)	0.400–6.400	0.260–0.860	Bone demineralization, skin rashes, hair depigmentation, decreased serum cholesterol, and increased alkaline phosphatase activity, increased premenstrual pain in women	(19, 20)
Iodine (I)	0.010–0.019	0.0003–0.0065	Goiter	(1, 21)
Selenium (Se)	0.001–0.013	0.000095–0.000225	Infertility, myodegenerative diseases, cardiovascular disease, and cognitive decline	(16, 22)
Molybdenum (Mo)	0.0055–0.0449	0.0290–0.130	Encephalopathy	(17, 19, 23)
Cobalt (Co)	0.001–0.00225	ND	Numbness, tingling in your hands and feet, and severe tiredness (fatigue)	(24)
Nickle (Ni)	0.120–2.530	0.020–0.350	Anemia and parakeratosis-like damage	(25)
Vitamin A (Retinol)	5.630–119.460	0.00462–0.818	Impaired immunity, rashes, and ocular effects, i.e., night blindness and xerophthalmia	(1, 19)
Vitamin D (Calciferol)	0.030–0.460	ND	Osteomalacia resulting in bone pain, muscular weakness, and weak bones	(1)
Vitamin E (α-Tocopherol)	0.300–11.030	0.00027–0.00443	Hemolytic anemia in infants	(16, 19)
Vitamin K (Phylloquinone)	0.0001–0.002	0.130–0.300	Poor bone development, bleeding, increased cardiovascular diseases, osteoporosis	(26, 27)
Vitamin C (Ascorbic acid)	140–9140	ND	Scurvy	(1)
Vitamin B_1_ (Thiamin)	0.120–0.900	0.020–0.117	Beriberi or thiamine deficiency	(16, 17, 19)
Vitamin B_2_ (Riboflavin)	0.010–0.220	0.016–0.220	Skin disorders, i.e., angular stomatitis, hair loss, cheilosis, sour throat	(16, 17, 19)
Vitamin B_3_ (Niacin)	1.700–8.200	0.526–1.060	Dementia, diarrhea, could often lead to death	(16, 17)
Vitamin B_5_ (Pentothenic acid)	0.050–0.950	0.0600–0.318	Insomnia, depression, irritability, vomiting, stomach pains, burning feet	(17, 19, 27)
Vitamin B_6_ (Pyridoxine)	0.00015–0.00050	0.039–0.134	Electroencephalographic abnormalities, swollen tongue, depression and confusion	(16, 19)
Vitamin B_7_ (Biotin)	0.0001–0.002	0.012–0.110	Alopecia and scaly erythematous dermatitis, aciduria, acidemia, hearing and vision problems, and reduce the growth	(27, 28)
Vitamin B_9_ (Folic acid, Folacin)	0.003–0.078	0.0036–0.067	Megaloblastic anemia having larger red blood cells than usual	(16, 19)
Vitamin B_12_ (Cobalamin)	0.020–0.450	0.0007–0.008	Anemia followed by damaged nerves, can affect thinking and memory	(1, 29)
**Macronutrients (g/100 g of food)**				
Carbohydrates	42.000–46.100	20.130–27.420	Siabetic ketoacidosis, hyperosmolar coma, and hypoglycemia ultimately affecting the central nervous system	(16, 30)
Proteins	7.900–9.400	7.550–9.020	Kwashiorkor, marasmus	(16, 30)
Fats	39.300–42.700	0.400–2.660	Scaly dermatitis, alopecia, thrombocytopenia, intellectual disability	(16, 30)
Potassium (K)	0.150–0.410	0.291–0.391	High blood pressure, muscle weakness, constipation, fatigue	(16, 17)
Calcium (Ca)	0.010–0.140	0.019–0.050	Muscles aches, spasms of muscles in the throat, tingling in different body parts, abnormal heart rhythms	(16, 17)
Magnesium (Mg)	0.020–0.120	0.036–0.057	Diabetes, coronary heart disease, hypertension, osteoporosis	(16, 17)
Sulfur (S)	0.08–0.160	ND	Obesity, Alzheimer's disease, chronic fatigue	(31)
Phosphorous (P)	0.289–0.1160.2	0.114–0.180	Anorexia, ataxia, confusion, anemia, proximal muscle weakness, skeletal effects, increased infection risk, paresthesias, confusion	(1, 17)
Sodium (Na)	0.001–0.0210	0.230–0.930	Brain swallowing is indicated by headaches, seizures, coma, and even death	(16, 32)
Histidine (HI)	2.000–3.800	12.000–19.370	Anemia and low blood levels	(16, 30)
Isoleucine (IIe)	3.000–4.200	20.000–30.620	Wasting and weakening of muscles and tremors	(16, 30)
Leucine	6.300–14.200	41.900–60.600	Poor growth, weight loss, rashes, hair loss, desquamation, poor feeding	(16, 30)
Lysine (Lys)	2.100–4.300	41.250–48.200	Red eyes, irritability, hair loss, anorexia, nausea, inhibited growth, fatigue	(16, 30)
Tryptophan (Trp)	1.000–2.500	1.875–6.900	Anxiety and irritability, aggressions, increased pain sensitivity	(16, 30)
Methionine (Met)	1.200–2.500	7.500–18.200	Tremor, low intelligence, abnormal muscle contractions, severe headache, abnormal eye developments	(16, 30)
Phenylalanine (Phy)	4.300–5.500	20.630–50.630	Behavioral problems, small head, seizures	(16, 30)
Threonine (Thr)	2.400–4.000	19.400–32.500	Lameness, neurological disorders	(16, 30)
Valine (Val)	3.600–6.700	23.200–32.500	Dehydration, hypotonia, loss of appetite, vomiting	(16, 30)
Linoleic acid	0.700–3.520	16.000–57.000	Poor growth, fatty liver, reproductive failures, skin lesions	(16, 30)
Linolenic acid	0.070–0.190	2.000–46.000	Intellectual disability, scaly dermatitis, alopecia, thrombocytopenia	(16, 30)

Most people depend on cereals and pulses to fulfill their dietary requirements. Around 60% of the calories and dietary requirements in the developing world and 30% in the developed world are extracted from cereals- and pulses-based food (38). However, cereal does not have a balanced nutritional profile, i.e., iron and zinc and other essential nutrients, which is the leading cause of malnutrition (Table 1). The population residing in developing countries is at high risk of malnutrition; more than 1 in 5 newborns did not receive a balanced diet and suffer from impaired cognitive development. More than 40% of children in South Asia and Africa are suffering from malnutrition. Approximately 1 in 14 children are wasted, 1 in 20 children are overweight, and 45% of deaths in <5-year-old children are associated with undertaking of a nutrient-poor diet. These facts are associated with undertaking a nutrient poor diet by a newborn and its lactating mother (39, 40). Half of the world's population uses white rice as a staple food due to its palatability, taste, and softness. However, its nutritional value is very low, it has a low concentration of vitamin E (0.0075–0.30 mg/100 g), niacin (1.3–2.4 mg/100 g), riboflavin (0.02–0.06 mg/100 g), thiamine (0.02–0.11 mg/100 g), fiber (0.2–0.5 g/100 g), fats (0.2–0.5 g/100 g), protein (6.3–7.1 g/100 g), and traces of Zn (0.3–2.1 mg/100 g) and Fe (0.2–2.7 mg/100 g). While brown rice has enough vitamin E (0.9–2.5 mg/100 g), niacin (3.5–5.3 mg/100 g), riboflavin (0.04–0.14 mg/100 g), thiamine (0.29–0.61 mg/100 g), fiber (0.6–1.0 g/100 g), fats (1.6–2.8 g/100 g), protein (7.1–8.3 g/100 g), and relatively high amounts of Zn (1.5–2.2 mg/100 g), and Fe (0.7–5.4 mg/100 g), which are essential for proper growth and development. Hence, it is strongly recommended to use brown rice to harvest maximum dietary benefits (41). Iron (Fe) is considered the most important element for health, with its deficiency leading to severe anemia. Similarly, deficiency of β-carotene and niacin leads to childhood blindness and pellagra (in which a person has wounds in the mouth), dementia, and diarrhea, respectively (9).

## What to Biofortify?

Biofortification ensures maximum uptake of minerals, their transportation to eatable parts, and bioavailability. There is an utmost need to biofortify cereals and pulses for essential micronutrients for the provision of balanced diets to the masses (5, 42). The following section briefly describes deficiencies of different cereals and pulses crops that need to be biofortified.

### Cereals

The commonly cultivated cereal crops include rice, wheat, maize, sorghum, barley, millets, and oats. Rice is reportedly deficient in Fe, Zn, and pro-vitamin A, which is a leading cause of hidden hunger in rice-eating areas around the globe and causes anemia, night blindness, and loss of vision (9). Wheat needs biofortification for effective provision of zinc, iron, selenium, grain yellow pigment contents (GYPC), pro-vitamin A, grain anthocyanins, and essential amino acids, similarly some anti-nutrient compounds, i.e., phytic acid, are present in excessive quantities which needs to be checked (43). Maize is found deficient in vitamin E (tocopherol, tocotrienol) along with persuasive antioxidants, vitamin C, vitamin A, anti-nutrient components, and quality protein (44). There is a need to enhance tryptophan and lysine content to increase nutritional quality of vital zein proteins. The opaque-2 (o2) mutant form in maize has the potential to increase tryptophan and lysine contents. Varieties possessing o2 allele have relatively high lysine and tryptophan contents as compared to wild typed varieties (45, 46). Micronutrients, i.e., zinc, iron, health-supporting γ-linolenic acid, polyunsaturated fatty acids, and stearidonic acid (STA), are also reportedly deficient in barley. Barley seeds have a phytase gene (*HvPAPhy a*) that is responsible for enhanced phytase action. This increased phytase activity improves zinc and iron bioavailability. Due to overexpression (Box 1) of cellulose synthase-like gene (*HvClF*), β glucans content increases. These are the dietary fibers involved mainly in the reduction of type II diabetes and cardiovascular disease (6). Sorghum is also reported to be deficient in iron and zinc along with iodine, selenium, magnesium, and calcium (47).

### Pulses

About 1,000 pulses are known to humans but only 20 of them are cultivated for human consumption including cowpea (*Vigna unguiculata*), pigeon pea (*Cajanus cajan* L.), chickpea (*Cicer arietinum* L.), urban (*Vigna mungo* L.), mung bean (*Vigna radiata* L.), French bean (*Phaseolus vulgaris*), lentil (*Lens culinaris* Medik), horse gram (*Macrotyloma uniflorum*), soybeans (*Glycine max*), moth bean (*Vigna aconitifolia*), Lathyrus (*Lathyrus sativus* L.), field pea (*Pisum sativum* L.), etc. (5). Pulses are deficient in iron and zinc contents like the majority of cereals (42). These are also deficient in selenium, dietary fibers, and sulfur-containing amino acids including cysteine and methionine (48). Although pulses are rich in proteins and micronutrients, there are some anti-nutrients as well which restricts the bioavailability of proteins and nutrients, e.g., lectins, phytic acid, saponins, lathyrogens, protease inhibitors, α-amylase inhibitors, and tannins, restrict the absorption of iron, zinc, calcium, magnesium, and other essential nutrients (49). There is a need to understand the metabolic pathway involved in the synthesis of these compounds and the disruption of key genes involved for their reduced production.

As we are talking about the removal of anti-nutrient compounds from cereals and pulses, we must also consider their vital functions in plant development as discussed below. **Lectins:** are a diverse family of plant proteins found in almost all organisms including animals, microorganisms, and plants, which restricts the bioavailability of iron and zinc. There are over 500 members of lectins in plants that are produced in response to biotic stresses, i.e., disease, insect, molds, and fungi attack and serve as the first defense line (50). **Oxalates:** or oxalic acid, are substances that can form insoluble salts with sodium, potassium, magnesium, iron, and calcium and restricts their bioavailability. These are produced in all photosynthetic organisms for calcium regulation, detoxification of heavy metals, and plant protection (51). **Phytate:** phytic acid or myo-inositol hexaphosphate (IP6) is another plant-derived anti-nutrient compound that restricts the bioavailability of iron and zinc. It usually serves as a storage house for plant phosphates, antioxidants for germinating seeds, and energy sources. These are usually produced during seed germination and serve as a source of 50–60% phosphorus during germination (52). **Tannins:** are a class of high molecular weight polyphenols that are found in the plant kingdom and serve as anti-nutrients by chelating iron, zinc, and copper. There are two classes of tannins, i.e., hydrolyzable tannins and condensed tannins. Condensed tannins are found abundantly in plants and play a role in plant defense as an antioxidant, anti-carcinogenic, immunomodulatory, detoxifying, and cardio-protective activities (53). Given the important metabolic activities of anti-nutrient compounds, there is a need to tradeoff between their desirable and undesirable functions before their silencing or knocking out (54).

## Strategies to Address Malnutrition

Many direct and indirect approaches are used to increase concentration of essential nutrients in grain and other parts of plants mainly to overcome malnutrition (55). Dietary diversification (Box 1), artificial supplementation (Box 1), and biofortification are used to overcome malnutrition. However, biofortification is cost-effective, feasible, and a long-term solution to reduce this nutrient deficiency. The biofortified crops have all the essential vitamins, minerals, and fatty acids that are required by a human to fulfill his or her nutritional demands (56–58). Furthermore, biofortified crops (Box 1) are eco-friendly as these enhance nutrient uptake from soil and improve soil health and these are sustainable as well (59, 60). For biofortification different agronomic, conventional breeding, transgenic, and gene editing approaches are used as described below.

### Conventional Approaches

**Figure 1:**
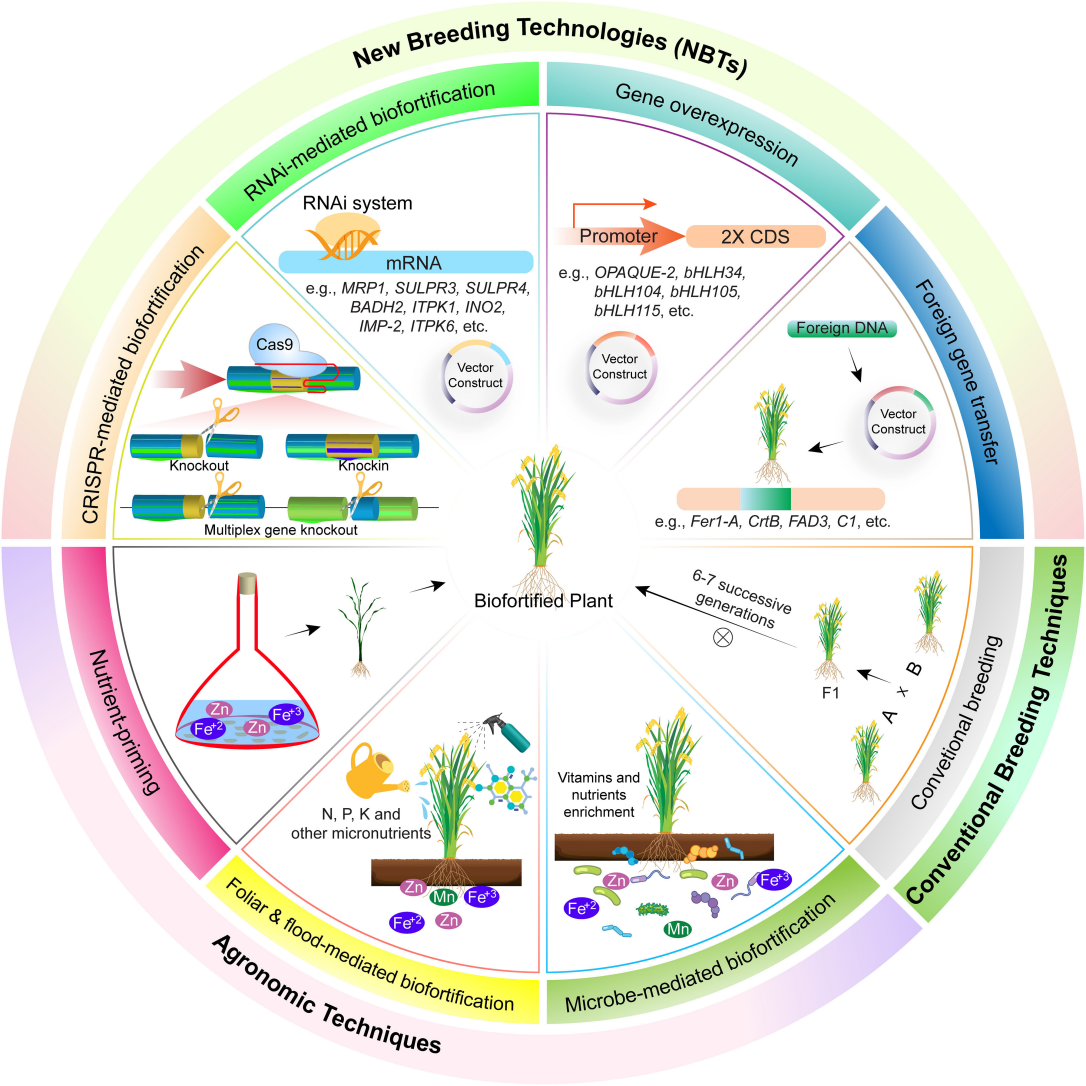
Different methods for biofortification of crops. These are broadly classified into three types, i.e., agronomic techniques, biofortification of crops through foliar spray of micronutrients. Foliar application helps in acquisition of more nutrients in reproductive parts, and hence healthier foods are delivered to the consumer. In this technique, nutrients are applied in liquid form in aerial parts of plants and got absorbed through stomata and epidermis and become part of the food chain. Mineral fertilization through flooding for biofortification of crops. Minerals, i.e., selenium, zinc, calcium, etc., are supplied to crops alongside irrigation and are readily available for uptake and as a result their accumulation in eatable parts of plants is increased. Mineral fertilization through soil application for biofortification of crops. Mineral fertilizers, usually NPK, are applied in the soil bed before sowing or alongside seed using different seed cum fertilizer drills and as a result they are absorbed and made part of the food chain through root uptake. Microbe-mediated enhanced uptake of nutrients for biofortification of crops. Different microbial species, i.e., rhizobium bacteria, mycorrhizae fungi, etc., help plants in nutrient acquisition through mutualism. Conventional breeding helps in biofortification of crops by crossing two parents possessing contrasting phenotypes and selection in subsequent segregation generations based on trait of interest. New breeding techniques, knocking out of genes involved in biosynthesis of anti-nutrient compounds. Different anti-nutrient compounds, i.e., lectins, phytic acid, saponins, lathyrogens, protease inhibitor, α-amylase inhibitors, and tannins restrict bioavailability of essential micronutrients. Hence, this results in malnutriated crops. Genes involved in biosynthesis of anti-nutrients could be repressed through RNAi for reduced accumulation of these compounds. Overexpression of gene responsible for micronutrient accumulation in plants leads toward micronutrient biofortification. Overexpression of genes leads toward increased micronutrients accumulation and, as a result, more deposition in the eatable plant parts. Biofortification of crops through gene transfer from other species has resulted in improvement of nutritional quality of crops and alleviation of malnutrition. Different genes involved in biosynthesis of pro-vitamin A (CrtB), iron homeostasis (Fer1-A), and flavonoids production (C1) has been transferred across species for biofortification. These genes help in the production of a balanced diet. As a result, different diseases associated with malnutrition are controlled, e.g., Golden Rice has helped to overcome night blindness and other diseases associated with pro-vitamin A deficiency. However, there are certain limitations of this technology, i.e., laborious, expensive, time consuming, and above all else, it has less public acceptability due to regulatory issues.

Biofortification through agronomic approaches is an economical and easy method but the method of nutrient application, their type, and environmental factors requires great care. This approach focuses on enhanced nutrient availability to the plants, their effective utilization, and mobility in plants, and an increase in microbial activity for their efficient utilization (Figure 1). Microbes like rhizobium, bacillus, azotobecter, actinomycete, and some fungal strains, i.e., *P. indica*, are used to enhance nutrient availability and uptake (61). Mineral nutrients also hold good promise for biofortification by soil and foliar application. The most used fertilizer is nitrogen, phosphorus, and potassium (NPK) based, which are essential for both plant and human health. Some other micro-minerals like iodine, copper, zinc, iron, nickel, manganese, molybdenum, etc. are also essential for crops. Most of these micronutrients are readily available in the soil and absorbed by plants and become part of the food chain. But sometimes plants are unable to absorb them and hence are applied in the form of mineral nutrition (62).

Apart from agronomic biofortification (Box 1), conventional breeding (Box 1) relies on the genetic variability for the trait of interest (TOI) in the gene pool (55). The desired genes are pyramided using the conventional crossing techniques followed by extensive screening of segregation populations. The Wheat Research Institute (WRI), Faisalabad and National Agriculture Research Council (NARC), Pakistan had released biofortified wheat varieties, i.e., “Zincol” and “Akbar-2019” back in 2015 and 2019, respectively, with enhanced iron and zinc contents (63). Similarly, Bangladesh Rice Research Institute (BRRI) released high iron and zinc rice varieties, i.e., Dhan 62, 64, 72, and Jalmagna (9). Many common bean varieties including MAC42, CAB2, RWV2887, 1129, 3317, 3006, and 33166, MAC44, RWR2154, PVA1438, HM21-7, CODMLB 3,2 and 001, RWR2245, and Cuarentino with high zinc and iron contents were released to reduce malnutrition. Some lentils verities Idlib3, Idlib2, Alemaya, L4704, Shital, Simal, Sisir, Khajurah2, ILL 7723, Barimasur 8, 4, 7, and 5 with high zinc contents were developed and released (55, 64). Further varieties developed through conventional breeding are summarized in Supplementary Table 1. Crop wild relatives (CWRs) harbor important genes that are the source of essential micronutrients. To achieve standard nutritional concentrations and protein requirements, the introgression of genetic variation from crop wild relatives to modern crops is inevitable (65). The most efficacious example of wild hybridization is the introgression of *Gpc-B1* locus in bread wheat (*Triticum aestivum*) from wild emmer (*Triticum turgidum* ssp. *dicoccoides*) *via* chromosomal substitution. This introgression was highly fruitful because it delivered wheat plants with an improved nutritional profile (Mn, Fe, and Zn) without yield penalty (66).

#### HarvestPlus Program

It is worthwhile to mention the contribution of the HarvestPlus program, which is working in connection with agriculture and nutrition to end micronutrient deficiencies and hidden hunger globally. It is targeting the most vulnerable communities and providing them a food-based solution to control micronutrient deficiencies. The organization is working alongside its partners to develop crop varieties that are rich in iron, zinc, and vitamin A, the three nutrients that are most lacking in global foods as identified by World Health Organization (WHO) (67).

It is working for the development of nutritionally enriched crops that could be evidence-based, cost-effective, and sustainable using conventional breeding approaches. The program is aimed at reaching 1 billion individuals with a supply of biofortified crops by 2030. HarvestPlus is currently focused in Pakistan, India, Bangladesh, the Democratic Republic of Congo, Latin America and the Caribbean, Nigeria, Rwanda, Uganda, Zambia, and Zimbabwe. The program was divided into three phases, i.e., discovery phase (2003–2008), which was aimed at the identification of high-risk populations, their food consuming habits, plant breeding resources, feasibility studies, and a pilot project to deliver pro-vitamin A enriched sweet potato in Africa. The development phase (2009–2013) was aimed at the evolution of biofortified crop varieties about iron, zinc, and pro-vitamin A in target countries, their testing at multiple locations for stability analysis, and researchers assessed the nutritional profile of developed varieties and country-wise teams were developed for delivery of biofortified crops. The delivery phase (2014-onward) was focused on the creation of consumer demands for biofortified crops to reach maximum populations. Researchers were working in the estimation of the area covered by biofortified crops and making efforts for its long-term sustainability (68).

Overall a total of 242 crop varieties has been released in 30 countries since the inception of HarvestPlus, 8.5 million smallholder farming households are growing these varieties and distributing them to 42.4 million people around the globe. The program was focused on the development of zinc biofortified wheat in Pakistan and rice in Bangladesh, vitamin A enriched cassava, maize, and iron enriched beans in the Democratic Republic of Congo, and iron and zinc enriched peal millet and wheat in India (https://www.harvestplus.org/where-we-work). The details of other countries along with success stories in the form of biofortified varieties evolved are summarized in Table 2.

**Table 2:** Summary of ongoing activities and achievement of HarvestPlus program.

**Country**	**Crop**	**Nutrient**	**Varieties developed**
Pakistan	Wheat	Zinc	Zincol-2015, Akbar-2019
Bangladesh	Rice	Zinc	BRRI dhan62, BRRI dhan64, BRRI dhan72, BU Aromatic Zinc Rice
Democratic Republic of Congo	Cassava	Vitamin A	Kindisa (TMS 2001/1661), Lumonu, Vimpi
	Maize	Vitamin A	PVA-SYN12F2 (composite), SAM 4 VITA (composite), GV605 (hybrid), GV604 (hybrid)
	Beans	Iron	HM 21-7 (bush), COD MLB 032 (bush), RWR 2154, RWR 2245 (bush), COD MLV 059 (bush), PVA 1438 (bush), Nain de Kyondo (climber), Namulenga (climber), Cuarentino (climber)
India	Pearl millet	Iron	ICMH 1201 (Shakti-1201), ICTP 8203-Fe-10-2 (Dhanashakti)
	Wheat	Zinc	BHU-6 (Chitra) and BHU-3
Latin America and the Caribbean	Cassava	Vitamin A	No data
	Beans	Iron	No data
	Maize	Zinc	No data
	Rice	Zinc	No data
	Sweet potato	Vitamin A	No data
Nigeria	Cassava	Vitamin A	TMS 01/1368—UMUCASS 36, TMS 07/0593—UMUCASS 45, NR 07/0220—UMUCASS 44, TMS 07/539—UMUCASS 46, TMS 01/1371—UMUCASS 38, TMS 01/1412—UMUCASS 37
	Maize	Vitamin A	Sammaz 37, 38, and 39, Ife Hybrid 4, Oba Super 6, SC 510, SDM4, Orange Maize, PVA 2 (Kapam 6)
Rwanda	Beans	Iron	RWR 2245 (bush), MAC 44 (climber), RWV 1129 (climber), RWR 2154 (bush), CAB 2 (climber), RWV 3317 (climber), MAC 42 (climber), RWV 3006 (climber), RWV 3316 (climber), RWV 2887 (climber)
Uganda	Bean	Iron	Roba 1 (bush)
	Sweet potato	Vitamin A	Ejumula, Vita, Kakamega, Naspot 12 O, Naspot 13 O, Kabode
Zambia	Maize	Vitamin A	GV662A (Kamano Seed), GV665A (SeedCo), GV664A, GV671A (ZamSeed), GV573 (Advanta), GV672A (Afriseed)
	Sweet potato	Vitamin A	Olympia, Chiwoko, Twatasha, Zambezi, Kokota, Mansa Red, Chingovwa
Zimbabwe	Maize	Vitamin A	ZS242, ZS246, ZS244, ZS248
	Beans	Iron	No data

*Note the information was obtained from the official website of HarvestPlus (https://www.harvestplus.org/)*.

### New Breeding Techniques

New breeding techniques (Box 1), i.e., transgenic breeding (Box 1), RNA interference (RNAi) (Box 1), and genome editing (Box 1), are playing key roles in the biofortification of crops by opening new avenues for the creation of novel genetic variation that does not exist in the gene pool as reviewed in Van Der Straeten et al. (69).

#### Transgenic Breeding

##### Cereals

Biofortification through transgenic breeding is an efficient, sustainable, and cost-effective approach to combat malnutrition. Transgenic crops (Box 1) are constituted when limited genetic variability is present in the gene pool and wild relatives. Transgenic breeding helps in biofortification through the introduction of genes that either decrease anti-nutrient compounds or increase micronutrients accumulation and bioavailability (Figure 1) (12). Detailed discussion about transgenic cereals and pulses developed for micro- and macronutrients (like vitamins, fatty acids, minerals, and amino acids) biofortification is summarized below.

The deficiencies of micronutrients like zinc, iron, vitamin A, and proteins in cereal grains could be overcome by the introduction and overexpression of genes corresponding to these micronutrients. For example, high iron accumulation in wheat grain was obtained through ectopic overexpression of *TaFer1-A* and *OsNAS2* genes which increased iron content up to 80 and 85 μg/g in grains, respectively, but *TaFer1-A* expression is not uniform across generations (71, 72). Similarly, overexpression of *TaVIT* doubled the iron contents in wheat grain; however, distribution was not uniform for whole grain (73). Overexpression of the *Amaranthus albumin-*based *Ama1* gene overcomes essential proteins and the amino acid deficiency (tyrosine, lysine, cysteine, and methionine) (74). Overexpression of the phytochrome (*PhyA*) gene enhanced phytase production and anti-nutrient activity, whereas silencing of *phyA* and *TaABC13* transporter gene decreased phytic acid production (18–19%) and increased iron and zinc bioavailability (75). The silencing of the *SBEIIA* gene increased concentration of less digestible amylose starch to combat obesity and over-nutrition (Box 1). The overexpression of carotene desaturase *CrtI, CrtB*, and *PSY* gene from bacteria also increased vitamin A concentration (73). A higher concentration of vitamin A, 4.96 μg/g DW, was attained after using maize *PSY1* gene encoding phytoene synthase and bacterial *Crtl* genes (76). *CrtB* or *Crtl* also resulted in increased accumulation of pro-vitamin A to as high as 3.21 μg/g of DW (77).

Among cereal crops, rice is biofortified with vitamin A, B1, B6, E iron, and zinc and other compounds (Table 3). Researchers indicated that vitamin A biofortification was done *via Phytoene synthase* (*PSY*) extracted from daffodil while phytoene desaturase (*Crtl*) genes extracted from *Erwinia uredovora* were later incorporated in *Agrobacterium tumefaciena*, which increased vitamin A concentration to 1.6 μg/g of DW (110). Another research highlighted that the *PSY* gene extracted from maize and *Crtl* from *Erwinia uredovora* also enhanced vitamin A concentration to 37 μg/g DW. Moreover, *PSY* and *lycopene* β*-cyclase* (β*-lcy*) extracted from daffodil also exhibited an increase in vitamin A concentration of around 1.6 μg/g DW (111–113) in rice. An overexpression of soybean ferritin gene *Soyfer H-1* gene resulted in an increase of Fe contents to as high as 38.1 μg Fe/g of DW, *Phaseolus ferritin* improving iron concentration to 22.07 μg Fe/g DW, *Osfer2* to 7.0 μg/g DW, while *OsNAS2* increased it to 19 μg/g DW (114). Moreover, overexpression of ferritin under control of endosperm-specific promoters' globulinb_1_ (*OsGlb*_1_) and glutelin B_1_ (*OsGluB*_1_), NAS with control of *OsActin1* promoter and *OsYSL2* along with *OsGlb*_1_ and *OsSUT*_1_ transport promoters resulted in iron biofortification in polished rice (115). Similarly, zinc biofortification with *HvIDS3, HvNAAT-A, HvNAAT-B*, and *HvNAS1* genes resulted in 35 μg Zn/g of DW (58), soybean ferritin, *Aspergillus flavus* phytase, *OsNAS1* exhibited 35 μg Zn/g of DW, while overexpression of *OsNAS2* resulted in 76 μg Zn/g of DW in rice crop (11). Another study highlighted the biofortification of endogenous rice lysine-rich histone proteins by overexpression of RLRH_1_ and RLRH_2_ with modified rice glutelin1 promoter. As a result lysine contents increased up to 35% in transgenic rice in comparison to wild type (116).

**Table 3:** Summary of studies explaining biofortification of crops through transgenic breeding and genome editing.

**Technique**	**Crop**	**Micronutrient**	**Genes involved**	**References**
Transgenic	*Triticum aestivum*	Iron	*TaFer1-A*↑, *OsNAS2*↑, *TaVIT2*↑, *GmFER*↑, *PhyA*↑, *NAM-B1*↑,	(12, 78)
		Tyrosine, lysin, cysteine, methionine	*Ama1*↑	(79)
		Vitamin A	*CrtI↑, CrtB↑, Bacterial PSY↑*	(77)
		Zinc	*OsNAS2↑, NAM-B1↑, PhyA↑*	(12, 78)
	*Oryza sativa*	Zinc	*IDS3↑, HvNAAT-A↑, HvNAAT-B↑, HvNAS1↑, OsNAS3↑, OsNAS1↑, OsNAS2↑, AtNAS1↑, AtIRT1↑, Pvferritine↑, Afphytase↑, OsFer2↑, OsHMA1↑, OsYSL15↑, OsYSL2↑, OSIRO2↑, OsVIT1↓, OsVIT21↓, OsYSL9↓*	(12, 80, 81)
		Iron	*AtNAS1↑, AtIRT1↑, Pvferritine↑, Afphytase↑, OsFer2↑, OsIRT1↑, OsNAS1↑, OsNAS2↑, GmFER↑, OsVIT1↓, OsVIT2↓, HvNAS1↑, OsYSL2↑, OsIDEF1↑, OsNAC5↑, OsYSL9↓*	(12, 80, 81)
		Vitamin E	*AtTC↑, AtHP↑*	(82)
		Vitamin B1	*THIC↑, THI1↑, TH1↑*	(83)
		Vitamin B6	*AtPDX1.1↑, AtPDX02↑*	(84)
		Vitamin B9	*ADCS↑, AtGTP cyclohydrolase 1↑*	(85)
		Provitamin A	*Carotene desaturase↑, daffodil PSY↑*	(80)
		Flavonoid, linoleic acid	*GmFAD3↑, ZmC1↑, chalcone synthase↑, phenylalanine ammonia lyase↑*	(86)
	*Zea mays*	Iron	*GmFER↑, aspergillus phytase↑, aspergillus phy2↑*	As proposed by (55)
		Lysine	*Sb401↑*	(77)
		β-carotene	*Zmpsy 1↑*	(87)
		Vitamin A	*crtI↑, crtB↑*	(77)
		Tocopherol, tocotrienol	*HGGT↑*	(87)
	*Sorghum bicolor*	Pro-vitamin A	*PSY-1↑, CRT-I↑, PMI↑, LPA-1↑↑*	(88)
	*Hordeum vulgare*	Zinc	*Zn transporter gene↑, DHPS↑*	As proposed by (55)
		Iron	*Phytase↑, AtZIP1↑*,	(12, 89)
		Lysine	*DHPS*	(89)
		Vitamin E	*AtVTE3↑, AtVTE4↑*	(90)
		β-glucans	*HvCs1F↑*	(90)
	*Cicer arietinum*	Iron	*GmFER↑, NAS2↑*	(91)
	*Phaseolus vulgaris*	Methionine	*Methionine rich storage albumin↑*	(92)
	*Glycine max*	Provitamin A	*Carotene desaturase↑, crtB↑, crtW↑, bacterial PSY↑, bkt1↑*	(93)
		Fe and zinc	*Phytase↑*	(12)
		Lysine	*Aspaktokinase↑, dihydrodipicolinic acid↑*	(94)
		Cysteine	*Maize zein protein↑, O-acetyl serine sulfhydrylase↑*	(94)
		Methionine	*Cystathionin γ-synthase↑, maize zein protein↑*	(95–97)
Genome editing	Rice	Fe	*OsVIT2↑*	(98)
		Amylose contents	*Wx gene↓ SBEI↓, SBEII↓, OsWaxy↓*	(99, 100)
		β-carotene	*OsCCD4a↑, OsCCD4b↑, OsCYP97A4↑, OsCCD7↑ and OsDSM2↑, Osor↑*	(12, 101)
		2 AP production and fragrance	*Badh2↑*	(100)
		Protein contents	*OsNRT1.1B↑*	(102)
		Phytic acid	*OsITPK1-6↓*	(103)
		Thiamine	*Ostpk1↑, Ostpk2↑, Ostpk3↑, Osncs1↑, OsThiC↑*,	As proposed by (104)
	Wheat	Fe and Zn	*TaVIT2↑*	(105)
		Grain weight	*TaGW2↑*	(106)
		Low gluten	*Alpha-gliadin 33-mer↓*	(107)
	*Maize*	Branched amino acids synthesis	*ZmALS1↑ and ZmALS2↑*	(108)
		Carotenoid synthesis	*ZmPSY1↑*	(109)

*Note:**↓** indicates the downregulation/knockout of gene, whereas **↑** indicates upregulation/overexpression of the targeted gene*.

Similarly, overexpression of rice genes *OsNAS1, OsNAS3, OsIRTI*, and *OsNAS2* accumulated double the amount of zinc. In rice *AtNAS1, AtIRT1, Afphytase*, and *Pvferritin* genes were also introduced to increase iron and zinc concentration and results were found satisfactory in polished rice. The disruption of activity of vacuolar iron transporter *OsVIT2* and *OsVIT1* gene also increased iron and zinc concentrations in rice grain (58). The overexpression of amino deoxy-chorismate synthase *ADCS* and *AtGTP-cyclohydrolase1* genes has enhanced folic acid content in rice (117). Golden Rice has high pro-vitamin A accumulation and it was considered the best example of biofortification using transgenic crops (118). The antioxidant activity in rice is linked with flavonoids that can be enhanced through overexpression of *chalcone synthase (CHS), maize C1, phenylalanine ammonia lyase*, and *GmFAD3* genes resulting in increased accumulation of flavonoids and linoleic acid accumulation in rice (119).

In maize endosperm, vitamin A accretion was increased by overexpression of bacterial *crtI* and *crtB* gene under the control of endosperm specific super gema zein promotor to as high as 9.8 μg/g of DW. Similarly, overexpression of maize *Psy1* gene also increased β-carotene accumulation in maize. Overexpression of the *dehydroascorbate reductase* gene increased vitamin C accumulation in maize by changing ascorbic acid oxidizing form into the reducing form (120). The overexpression of *aspergillus phytase, aspergillus niger phy2*, and *soybean ferrite* gene in maize enhanced iron bioavailability. The overexpression of the potato *sb401* gene enhanced lysine contents in maize grain. Antisense dsRNA targeting α-zeins enhanced tryptophan and lysine accretion in maize. The overexpression of the *Homogentisic acid Geranyl Geranyl Transferase* (*HGGT*) gene enhanced tocopherol and tocotrienol concentrations in maize (55) and resultantly biofortified maize was obtained. Furthermore, overexpression of *PSY1* maize also confirmed a higher concentration of vitamin A 59.32 μg/g of DW (121, 122).

Sorghum transgenic line homo188 harbors different genes, i.e., *PSY-1, CRT-I, PMI, LPA-1*, for enhanced pro-vitamin A content and has high nutritional value (88). Through overexpression of zinc transporter genes in barley, the amount of zinc was enhanced. The expression of *DHPS* and phytase gene causes an increase in lysine iron and zinc bioavailability in barley. The co-expression of the 2-methyl-6-phytyl benzoquinol methyl transferase *AtVTE-4* and *AtVTE3* gene reportedly enhanced vitamin E in barley seeds. Likewise, the expression of the *D6D* gene enhanced stearidonic acid and linolenic acid content in barley, which are essential for human health. The concentration of dietary fibers like β-glucans was also escalated barely through the overexpression of the cellulose synthase-like (*HvCslF*) gene (6). Transgenic millet reportedly exhibited higher zinc content owing to zinc transporters, while transcriptomics indicated higher calcium sensor genes involved in uptake, translocation, and accumulation of calcium in finger millet. Moreover, anti-nutrients including phytic acid, polyphenols, and tannins are also reported to have a lower concentration in the transgenic millet arisen through genome editing (123).

##### Pulses

Transgenic breeding was less explored in pulses for biofortification and few pulses like chickpea, common bean, soybean, and lupines are modified to overcome malnutrition (124). The basic aim of biofortification of pulses through transgenic breeding is the enrichment of essential amino acids, iron and zinc fortification, and reduction of anti-nutrients compounds. The deficiency of sulfur-rich amino acids was overcome through overexpression of heterologous proteins rich in these amino acids. Maize-based 27 kDaγ-zein, a cysteine-rich protein, was introduced and overexpressed in different pulses crops for nutritional enhancement of cysteine amino acid (48). Similarly, methionine concentration was enhanced in narbon bean and lupin by overexpression of S-rich proteins. The concentration of methionine was enhanced in Brazil nut by overexpression of the 2S albumin storage protein and aspartate kinase using seed-specific promotor and 4 times more methionine accumulation was achieved in seed (48). The rice *OASA1D* transgene enhanced free tryptophan accumulation in adzuki bean upon transformation (48).

Glycine max ferritin and chickpea NAS2 genes were introduced and overexpressed to increase iron bioavailability in chickpea (91). In soybean overexpression of cystathionine γ-synthase gene enhanced methionine concentration. Overexpression of maize zein protein in soybean enhanced cysteine and methionine content in seeds. The overexpression of the O acetyl serine sulfhydrylase gene also enhanced cysteine content in seeds. In transgenic soybean upregulation of dihydrodipicolinic acid synthase and aspartokinase gene enhanced lysine content in seeds (55). Overexpression of carotene desaturase and bacterial *PSY, bkt1, crtW*, and *crtB* gene enhanced pro-vitamin A accretion in soybean (125). The silencing of the ω-3 FAD3 gene was accomplished using siRNA-mediated knockout to reduce α-linolenic concentration in soybean. Similarly, the Δ6-desaturase gene was overexpressed to increase α-linolenic conversion to its stable form γ-linolenic acid and ω-3 fatty acids. Production of isoflavone in soybean was enhanced by maize C1 and R transcription factors drove gene activation (3, 55). The methionine contents in common beans were enhanced by the transformation of methionine-rich storage albumin from Brazil nuts. Similarly, the S-rich amino acid profile of lupins was improved by the transformation of the respective gene from sunflower and albumin (55). Due to space limitation, most transgenes are not discussed in detail and are summarized in Table 3.

#### RNA Interference

RNAi is a sequence-specific gene regulation process driven by a double-stranded RNA (dsRNA) molecule, which results in inhibition of either transcription or translation of a particular gene. Since its discovery, RNAi has opened a new vista for crop improvement. It is a precise, stable, efficient, and better tool than antisense technology. RNAi provides a platform for the incorporation of biotic and abiotic stress tolerance and delivery of quality food through biofortification and bio-elimination. It is widely used for nutritional quality enhancement of crops and removal of food allergens and contaminants (126, 127). The following sections cover some examples of the use of RNAi for the biofortification of cereals and pulses.

Phytic acid (PA) is considered a major anti-nutrient in cereals and pulses due to its ability to chelate micronutrients and restrict the bioavailability of important nutrients. RNAi was employed to disrupt an important gene [*inositol pentakisphosphate kinase* (*TaIPK1*)] of the PA biosynthesis pathway in wheat. The resultant homozygous transgenic lines of wheat at the T_4_ stage show reduced expression of *TaIPK1*, reduced PA accumulation (26–58%), and increased grain phosphate, zinc, and iron contents (128). Carotenoid contents have high nutritional value being precursors of pro-vitamin A. In rice, carotenoid contents were enhanced by RNAi-based disruption of carotenoid-cleavage dioxygenases (CCDs) genes which degrade carotenoids. Three genes, i.e., *OsCCD1, OsCCD4a*, and *OsCCD4b*, were disrupted using RNAi. Resultantly an increase in carotenoid production was observed in mutants, i.e., *OsCCD1-RNAi* (1.4-fold), *OsCCD4a-RNAi* (1.6-fold), and *OsCCD4b-RNAi* (1.3-fold) as compared to wild types (129). Lysine is the main limiting essential amino acid in rice, which serves as a source of energy and nutrition. Two enzymes, i.e., aspartate kinase (AK) and dihydrodipicolinate synthase (DHDPS), are liming factors for lysine and are extremely sensitive to a feedback mechanism. RNAi-based repression of AK and DHDPS enzymes was carried out in rice and resultantly 6.6- and 21.7-fold more lysine was accumulated in mutant lines, respectively. When both mutations were combined in one genotype, the lysine level was 58.5-fold more in wild types (130). Maize endosperm is deficient in pro-vitamin A and β-carotene leading to vitamin A deficiency in masses. However, other carotenoids, i.e., zeaxanthin which is produced from β-carotene *via* a two-step hydroxylation reaction, are found in sufficient quantity which may be stopped to increase β-carotene content in maize endosperm. Considering the abovesaid phenomena two maize genes *ZmBCH1* and *ZmBCH2* involved in hydroxylation reaction were knocked out using RNAi to enhance β-carotene in maize endosperm. Mutants for *ZmBCH2* genes showed a significant increase in β-carotene contents in maize grain indicating that this gene has a key role in conversion of β-carotene to other carotenoids (131). Due to the presence of γ-kafirin, sorghum was considered less digestible and through suppression of γ*-kafirin 1A*, γ*-kafirin1, 2*, and γ*-kafirin* through RNAi silencing its digestibility was improved (55).

Although the role of RNAi in pulses biofortification was not elaborated that much, potential also exists for improvement of pulses. Phytate restricts the bioavailability of micronutrients in pulses, studies have reported the complex formation of phytic acid with calcium, magnesium, copper, and iron, thereby reducing their solubility properties (4). Some other compounds, i.e., prebiotics mainly inulin and fructans, absorb iron, zinc, and calcium in themselves and restrict phytic acid activity. Similarly, β-carotene also promotes the absorption of iron and zinc in lentils, peas, and chickpeas. Thereby genes encoding these compounds could be overexpressed for enhanced micronutrient bioavailability (132). Selenium is likewise reported to enhance the bioavailability of iodine in lentils, peas, and chickpeas. However, some inhibitors preventing bioavailability need further investigation. The biosynthetic pathways involved in the production of anti-nutrients should be studied and genes that play a key role should be silenced or knocked out using RNAi for the development of nutritionally enriched pulses crops (42).

#### Genome Editing

**Figure 2:**
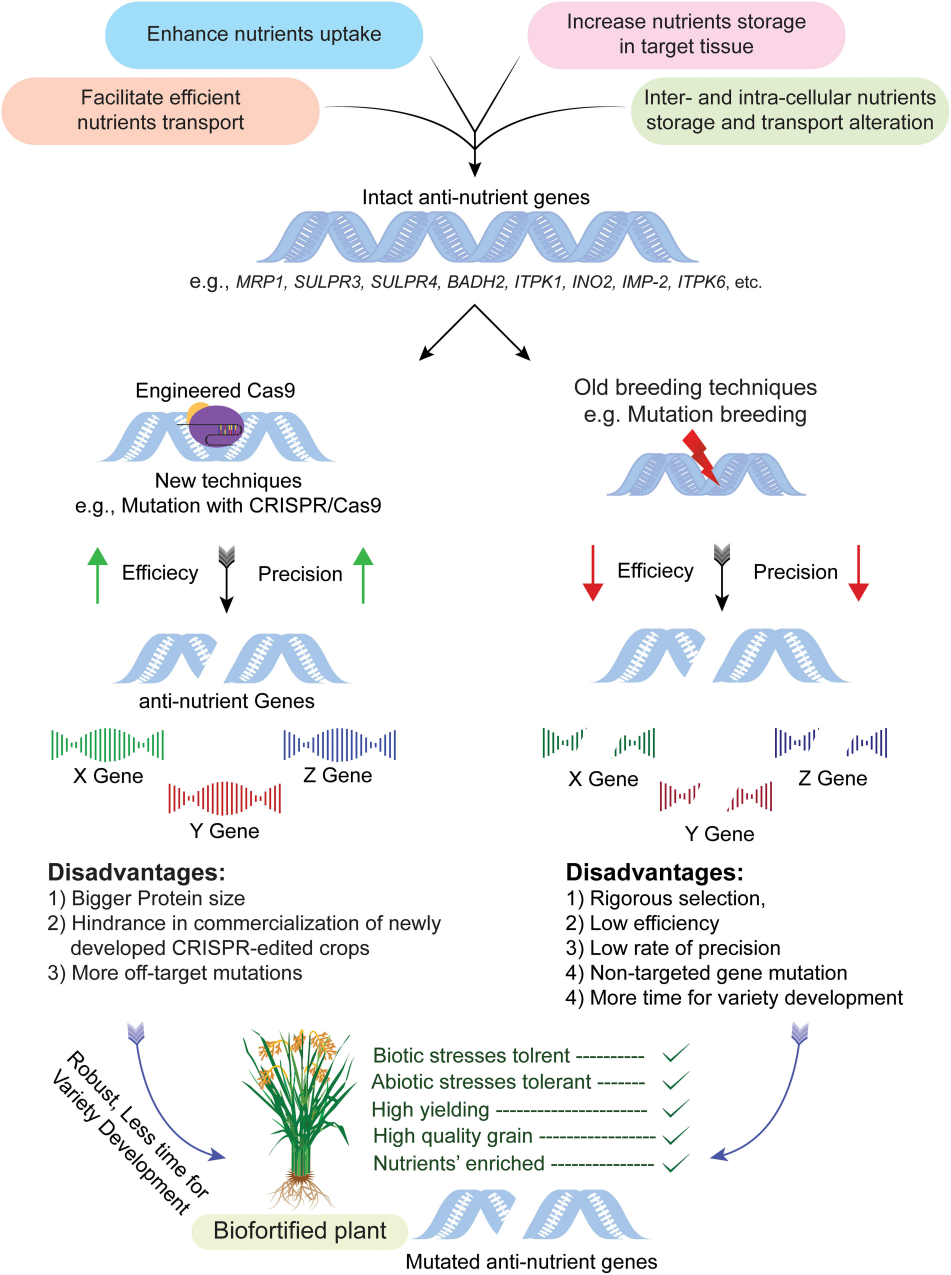
CRISPR-Cas system mediated targeted genome editing for biofortification of crops. The process begins by selection of cultivar having anti-nutrient genes, i.e., *MRP1, BADH2, INO2*, etc., followed by CRISPR/Cas system mediated modification without disturbing the genetic makeup of the rest of the plant and resultant mutants are obtained in a short period of time as compared to traditional mutagenesis. The mutant plants obtained through this technique are used in a breeding program for the development of nutritionally improved and transgene free varieties. The illustration also explains pros and cons of genome editing in comparison to old breeding techniques, i.e., the efficiency of conventional breeding is very low as compared to genome editing. Similarly, off-target effects of traditional mutagenesis are very high as compared to genome editing.

Sequence-specific nucleases (*SSNs*) are used in plant genome editing (GE) for stably inherited and targeted gene modification in the desired crop to produce transgene-free plants. Various types of SSNs, i.e., *TALENs, ZFNs*, and CRISPR*-*Cas system are used for plant genome editing (133). Genome editing through CRISPR involves Cas9/13, RNA-guided DNA endonucleases guided by a short guided RNA (sgRNA) resulting in a complex at the target site for targeted gene editing (Figure 2) (127, 134). Genome editing was exploited less for biofortification of cereals and pulses; however, some highlighted examples are discussed below.

##### Cereals

The rice *OsVIT2* gene was knocked down for increased Fe availability through genome editing (98). CRISPR-Cas9 was used for targeted insertion of 5.2 kb carotenogenesis cassette comprising of *Ctrl* and *PSY* genes of maize driven by endosperm specific promotor in the rice line Kitaake. The resultant mutants accumulated 7.9 μg/g dry weights (DW) β-carotene in endosperm, which is comparable to the Kaybonnet rice variety developed through traditional transgenic breeding commonly known as Golden Rice2 (135). Five rice carotenoid catabolic genes (*OsCCD4a, OsCCD4b, OsCYP97A4, OsCCD7*, and *OsDSM2*) were simultaneously mutated for enhanced β-carotene accumulation in rice endosperm; however, no satisfactory results were procured (101). Multiplex genome editing was performed by targeting the *OsWaxy* gene at three sites for reduction of AC in rice and 14% less accumulation was observed in mutants as compared to wild type (136). The nitrogen transporter gene *OsNRT1.1B*, which is linked with protein accumulation in rice, was edited for an increased uptake of nitrogen (102). CRISPR-Cas9 knock out of *OsITPK1-6* leads to low phytic acid accumulation in rice grain and resultantly increases micronutrient availability (103).

Zinc concentration was increased in wheat through targeted mutagenesis of the *TaVIT2* gene *via* genome editing (137). Four genes belonging to the *Alpha-gliadin* gene family in wheat code for high molecular weight gluten (HMWG) protein. Genome editing was also exploited for the biofortification of maize in different regulatory pathways. *Acetolactate synthase* genes (*ZmALS1* and *ZmALS2*) were edited for the accelerated synthesis of branched-chain amino acids, i.e., aline, leucine, and isoleucine (108). The phytoene synthase gene (*ZmPSY1*) involved in carotenoid biosynthesis pathway was modified using the CRISPR-Cas9 type II system and stable transformation was observed in progeny; however, functional characterization of mutants for carotenoid concentration remained in progress (109).

##### Pulses

Currently, the literature is deficient in examples explaining the role of genome editing for the biofortification of pulses. However, the potential exists in the exploitation of genome editing for iron and zinc biofortification; carried out by manipulation of iron-regulated transporter (IRT), ferric-chelate reductase oxidase (FRO), YELLOW STRIPE 1-like (YSL), natural resistance-associated macrophage protein (NRAMP), zinc-regulated transporters, and iron-regulated transporters like protein (ZIP) for an increased iron and zinc uptake in all pulses crops (124).

Another approach employed for the biofortification of pulses genome editing is to target anti-nutrient genes which are responsible for the reduced bioavailability of micronutrients. Saponins are the anti-nutrient compounds that—in lower concentrations—are beneficial; however, if consumed in higher quantities, they can act as anti-nutrients (42). An *Arabidopsis thaliana*-based study indicated that 13 OSC genes, 246 P450 genes, and 112 uridine diphosphate glycosyltransferases (UGTs) are involved in the biosynthesis of saponins. There is a need to identify key regulatory genes in the saponins biosynthesis pathway and eliminate them for reduced saponins production (138). Similarly, genes responsible for the production of other anti-nutrients, i.e., lathyrogens, protease inhibitor, and α-amylase need to be identified and consequently modified for reduced production of anti-nutrient compounds (4).

## Role of Genome-Wide Association Studies in Biofortification

**Figure 3:**
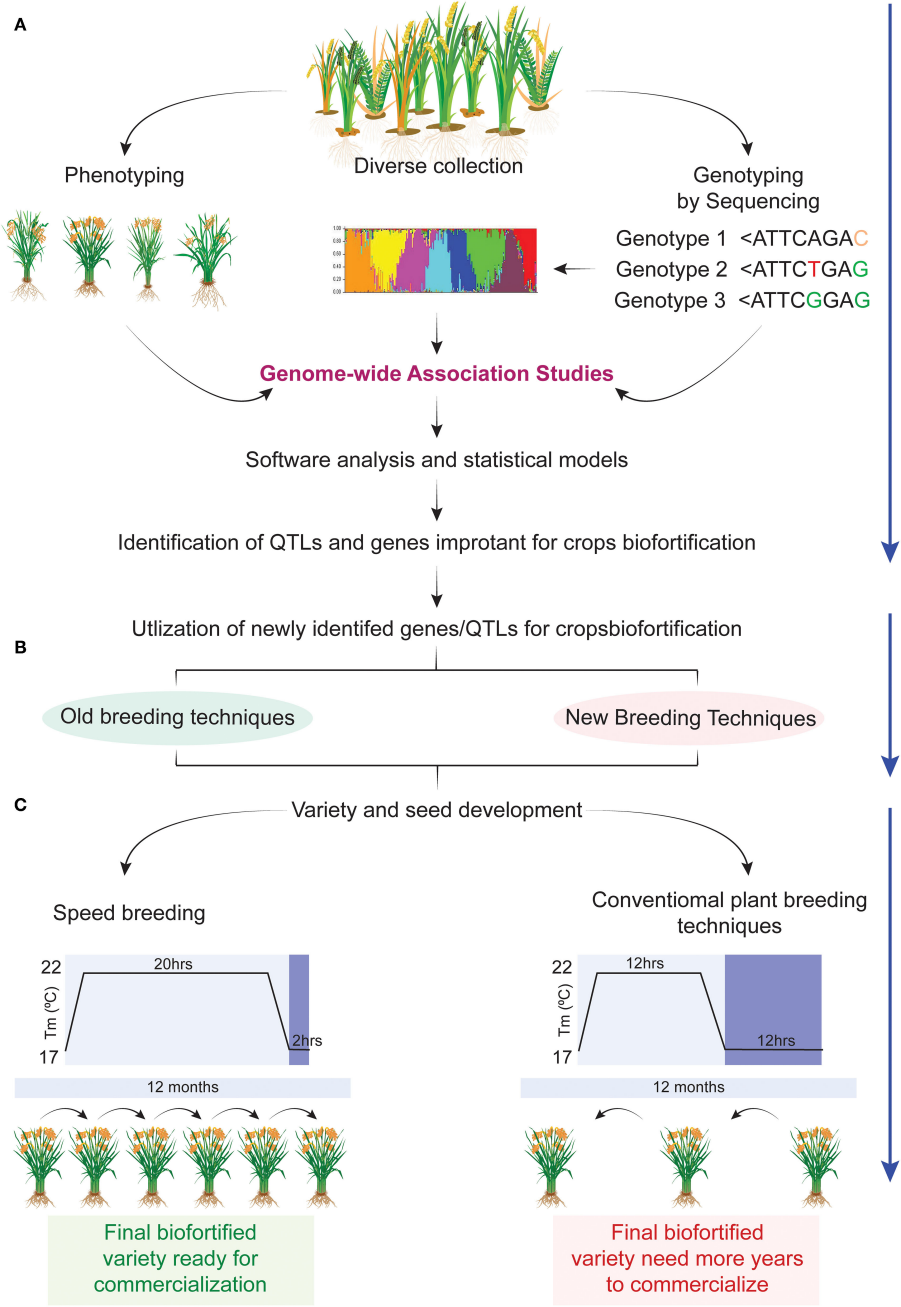
Speedy development of biofortified crops using next generation technologies. **(A)** Diverse collection of germplasm, its phenotyping and genotyping for nutrient profiling at various locations followed by genome wide association studies (GWAS) for identification of candidate genes involved in the mobility, accumulation, and partitioning of nutrients in various parts of plants. **(B)** Newly identified candidate genes in the previous step will be utilized in different breeding programs using the following breeding methods such as marker assisted breeding (MAB), gene pyramiding, transgenic breeding, and CRISPR-based gene editing for developing nutrient enriched crops. **(C)** Biofortified plants identified in the previous step can be continued for variety development using one of the two methods, i.e., conventional breeding or speed breeding. Conventional breeding will take double the number of years to achieve homozygosity in a calendar year as compared with speed breeding. In this way speed breeding will reduce the variety development span (70).

Understanding the genetics of complex traits is fundamental to developmental biology. Plant scientists were always curious to examine trait variation under different genetic backgrounds which have laid the foundation of different association studies. Continued progress in genome sequencing technologies, development of high density genotyping arrays, and high throughput phenotyping platforms paved the way for genome-wide association studies (GWAS) (139). GWAS starts with genotyping using different next-generation sequencing tools, i.e., genotyping by sequencing (GBS) or whole-genome sequencing (WGS) followed by extensive phenotyping under a given set of environments. The genotyping data are trimmed to obtain meaningful SNPs which are subjected to GWAS separately for each trait for the development of SNPs-trait association. As a result, candidate genes controlling particular traits are predicted (140) (Figure 3).

The advent of GWAS has opened new avenues for crop improvement by helping scientists in the identification of genes controlling complex phenotypes. Different GWAS studies were conducted in cereals and pulses for the identification of genes involved in micronutrients accumulation as detailed below. GWAS was conducted in maize for the identification of genes involved in the carotenoid biosynthesis pathway. For this purpose, 380 genotypes of maize from the CIMMYT carotenoid association mapping panel were used alongside 476,000 SNPs markers. The key genes involved in carotenoid biosynthesis pathways in maize, i.e., *DXS1, GGPS1*, and *GGPS2* which take part in the accumulation of precursor isoprenoids and downstream genes *HYD5, CCD1*, and *ZEP1* which play a role in hydroxylation and carotenoid degradation, were identified (141). GWAS was conducted in wheat with 35,648 SNPs and 123 wheat genotypes for identification of genetic regions associated with 10-grain minerals, i.e., Cd, Cu, Ca, Fe, Co, Li, Mg, Mn, Ni, and Zn. Ninety-two SNPs trait association were observed among which 60 were novel and 40 were within genes. Functional annotations of 20 genes out of 40 depicted their role in grain mineral accumulation. The majority of SNPs were identified from D-genome suggesting its role in controlling grain mineral diversity in wheat (142).

GWAS was used to understand the genetic architecture of seed molybdenum (Mo) and selenium (Se) in wild and cultivated chickpea. For that purpose, 180 individuals including 107 wild (*C. reticulatum*) and 73 cultivated (*C. arietinum*) were surveyed using 121,840 SNPs markers and phenotyped at two locations for 2 years. Sixteen SNPs were found associated with seed Mo and Se contents in chickpea, therefore recommending GWAS as a suitable technique for studying the genetics of complex traits (143). Similarly, a set of 174 accessions of Croatian common bean land races were phenotyped for seed contents of eight micronutrients (Mn, Zn, Fe, Mg, Ca, K, N, P) and genotyped using 6,311 high-quality diversity array-derived SNPs markers. GWAS identified 22 quantitative trait nucleotide (QTNs) for grain nitrogen content, 5 for phosphorous content, and 1 for calcium were identified whereas no QTNs were observed for other micronutrients (144).

## Role of Speed Breeding in Speedy Development of Biofortified Crops

The major bottleneck to the different breeding programs is the length of the breeding cycle of crops. After selection of parents and their intermating 4-6 generations are required to generate genetically stable homozygous lines for field evaluation, which takes a further 2–3 generations and resultantly breeding cycles span around 6–7 years for mono season crops and 3–4 years for two season crops (145). This creates demand for novel technologies to accelerate crop growth and reduce breeding time. Breeders are already using different strategies, i.e., double haploid and shuttle breeding to cut the short breeding cycle of crops (146, 147). However, these methods have their limitations and speed breeding (SB) is emerging as a novel tool for and attracting different breeders. This method was first proposed by the University of Queensland in 2003 as a combination of different methods to accelerate the breeding of wheat. The working principle of this method is a modification of the environment in such a way as to induce early flowering and reduce generation time (148). It applies to diverse germplasm and does not require tools for *in vitro* culturing and moving across the country to find a suitable climate for obtaining multiple generations in a year as required for double haploid and shuttle breeding, respectively (148) (Figure 3).

It is a well-established fact that plant growth is affected by many internal and external factors, i.e., temperature, photoperiod, light intensity, planting density, and light quality. SB modifies these natural processes and hijacks different biological processes of plants for rapid generation enhancement. Knowledge of these fundamentals processes is necessary for the development of effective SB platforms for a specific crop (148). Photoperiod plays a crucial role in the transition to reproductive development by sensing any change in the external environment as detected by photoreceptors. As a result, the reproductive success of a species is increased after synchronizing with the change in external stimuli. The plants are categorized into three classes, i.e., short-day plants (SDPs) which require longer than critical night length, long-day plants (LDPs) which are shorter than critical night length, and day-neutral plants (DNPs) which are not regulated by night length for triggering of flowering (149). Similarly, atmospheric temperature, light intensity, and planting density have critical roles in the modification of the reproductive development of a plant. These external stimuli are modified in a precise way for obtaining 4–5 generations of a species in 1 year at the same place (145). It has been effectively used in many kinds of cereal and pulses for shortening of breeding cycles, i.e., wheat (150), rice (151), peas (152), chickpea (153), and many other crops. The above-mentioned facts revealed that after modification of different genes/plant process using the abovesaid NBTs followed by rapid generation enhancement using speed breeding promise to deliver biofortified crops to consumers in shorter possible times. This section has also highlighted that rapid generation enhancement like speed breeding is inevitable in the days to come to ensure food security.

## Regulatory Aspects of Varieties Developed Through NBTs

The knowledge of genomics and its implementation in plant breeding has dynamically increased the use of modern plant biotechnology to improve the quantity and quality of crops. New plant breeding techniques (NBTs) are precise and accurate in obtaining desired mutants with high target specificity. Through these techniques, we can improve crops directly by deletion or insertion of a specific segment of a gene and ultimately obtain desired plant traits without affecting other characters in a cost-effective and time-efficient manner. These NBTs can be distinguished from other GM crops due to their stable and definite mutation. Despite its effectiveness, there is controversy in many countries about its usage. Some countries like China, Canada, Australia US, Brazil, and others are trying to adopt these techniques as advanced conventional breeding while other countries like the E.U. are uncertain about their adoption and regulation (154).

In the U.S. and the E.U., there are different policies for the production and consumption of these genetically modified crops. The main cause is people's perception of these products. Americans have a high acceptance of agriculture biotech products while not Europeans who fear the unpredictable risk of genetically modified crops (15). The regulatory authorities, scientists, and policy makers are talking about the genome-edited crops regulations. Their main point of discussion is whether to put genome-edited crops under the regulatory framework of GMOs. They are recommending it on the character trait being improved, different pathways adopted for improvement, and tools that are used, or the possible risks of crop end-products for classification of genome-edited crops.

**Figure 4:**
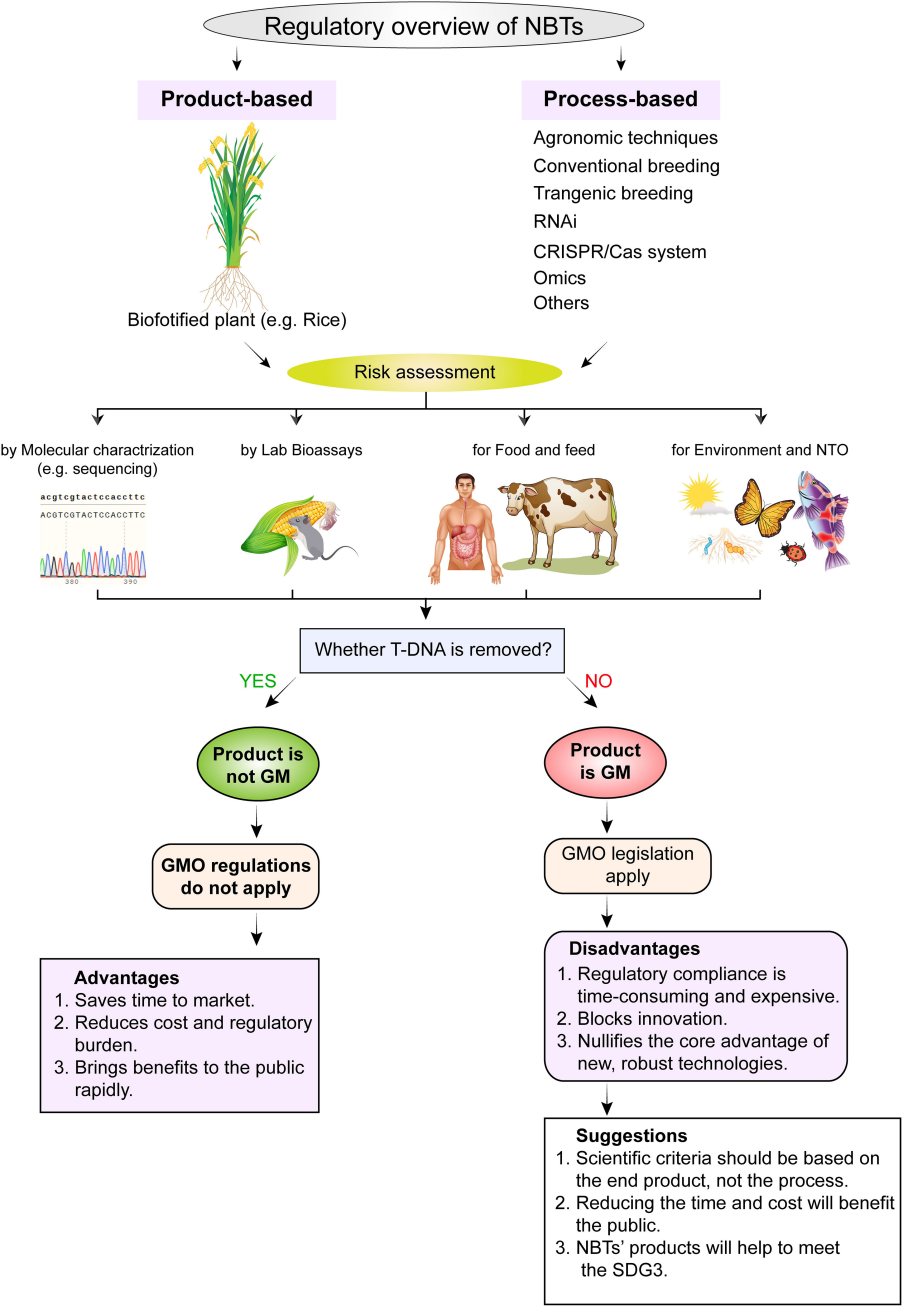
Regulatory framework and risk assessment strategies for commercialization of biofortified crops developed through new breeding techniques (NBTs). The regulatory framework consists of two methods, i.e., process-based regulation and end-product-based regulation. Whereas, risk is assessed by molecular characterization, lab bioassays for food and for environment and NTOs. NTO, non-target organism; T-DNA, transfer DNA; GMO, genetically modified organism; SGD3, sustainable development goal 3.

In 2012 the United States Department of Agriculture announced that mega-nucleases and ZNFs (Box 1) edited plants should not be placed under GMO regulation and allowed the commercialization and cultivation of waxy corn and CRISPR edited mushrooms without passing through GMO regulation (Figure 4). The U.S. regulation is product-based while the E.U. is process-based. However, some anti-GMO agencies are trying to ban genome-edited crops under GMO regulation using an unreasonable argument stating that these products are unnatural and they may affect the environment (15). It has been ordered by the European court of justice to put CRISPR edited crops under GMO regulation which has complicated the commercialization and trade relation of the European and other countries' markets. Therefore, the dependency of technology adoption and its success is based on not only the evidence and scientific methods but also the non-government agencies, regulators, and consumers' acceptance (127, 134, 154).

For transgenic crop cultivation and commercialization, different regulatory processes are time-consuming and expensive with low acceptance from people toward these products. For example, the development of Golden Rice was a great achievement in the field of biotechnology, but many years have passed and it is not ready for commercial cultivation because of its unstable yield. Because of this conflict, government has not approved its cultivation. Similarly, the cultivation of *Bt* brinjal was also banned because some anti-GMO agencies, scientists, and farmers showed concern about its cultivation. But later on, after several tests, four more varieties were approved and released for commercial cultivation. Therefore, the approval of this technology is associated and based on public acceptance (55).

## Conclusion and Future Perspectives

The United Nations (UN) is pushing member countries to meet the 17 set SDGs by 2030. Out of these, SDG3 is about ensuring healthy lives and promotes well-being for all at all ages. Good health is tightly linked with nutrition and consumption of nutritious food. However, hidden hunger is a major hurdle in ensuring good healthy lives by severely affecting the global population. Most vulnerable to this issue are young children and women in developing countries. The stagnation in the reduction of global micronutrient deficiency is owing to the widespread use of cereals around the globe resulting in an imbalanced nutritional profile. Pulses along with cereals meet complete dietary requirements and offer a balanced diet if consumed in the prescribed quantities (155). The biofortification of crops apart from offering balanced nutrition to masses also helps governments to achieve SDG3 by developing nutritionally enriched crops. Effective biofortification programs should aim at the development of crop varieties possessing enhanced micronutrient content without compromising economic benefits to farmers (58). The efficiency of an effective breeding program for biofortification is based on the availability of genetic diversity, reduction of anti-nutrients, and increased concentrations of promotor's substances, i.e., amino acids (methionine, lysine, and cysteine) and ascorbic acid (vitamin C) that can enhance absorption of essential elements (65).

There are certain limitations to the biofortification of crops through conventional and molecular means. For example, in conventional breeding, lack of genetic diversity in the gene pool is a major limitation which in some cases may be overcome by crossing with distant relatives for the introduction of trait of interest (TOI). However, in most cases, it is difficult to find the desired trait in distant relatives. Hence, it is almost impossible to improve that trait through conventional breeding, i.e., improving Se contents in wheat and improving oleic and linoleic acid in the soybean (156, 157). The unavailability of TOI in a species and its wild relatives is overcome through transgenic breeding. However, transgenic crops have their limitations, i.e., low consumer acceptability and strict regulatory approval process in various geographies. GM technology is inexpensive and time-consuming and is not among the good books of the current political and economic landscape. The success rate of the GM technology for cultivar released is very low irrespective of untiring efforts for gene identification, modification, expression in the target organism, agronomical evaluation, and biosafety assessment studies (55). As an example, we may discuss Golden Rice which was first developed and the report was published in 2000 and after 21 years of effort, the product is now approved for commercial cultivation in the Philippines (https://www.irri.org/news-and-events/news/philippines-becomes-first-country-approve-nutrient-enriched-golden-rice) due to yield barriers and consumer preference (118).

Genome editing offers a solution to most of the abovesaid problems. It is very precise and effective in inducing targeted modification in the gene of interest and is biologically safe as the end product is free from transgene (133). The latest breakthrough in genome editing, i.e., base editing (Box 1) (the irreversible conversion of a base at the target site without involving donor templates, double-stranded breaks, and dependency on NHEJ and HDR), prime editing (Box 1) (the introduction of indels and all 12 base to base conversions without inducing a DNA double-strand break using prime editing guide RNA (pegRNA) to drive the Cas9 endonuclease) and genome editing using rice zygote (which overcomes the problem in the delivery of macromolecule to the host cells and tissues and difficulty in transformation and regeneration) has opened new horizons for biotechnologists (158). As far as its regulation is considered, the U.S. and Canada have already declared the GE crops free from the GM regulations; however, debate is still ongoing in the E.U. with expected positive outcomes (127, 159, 160). GE crops are free from transgene, hence these should be regulated like conventional breeding products globally and should reach a maximum consumer for obtaining maximum benefits of technology in a shorter possible time (127).

The future of biofortification lies in genome editing. The targeted area that needs to be focused on is the engineering of genes for increased uptake of the micronutrients from the soil, maximum translocation to the seed, and increased bioavailability. Cereals and pulses offer a balanced diet if biofortified for Fe, Zn, sulfur-rich amino acids, and knocking out of anti-nutrient genes (161). The other area that needs special attention is engineering crops for reduced accumulation of anti-nutrients in cereals and pulses. The highlighted example in this context is PA contents in rice. PA is the most abundant storage form of phosphorous in plants which chelates metal ions and gets converted to phytate, and hence act as an anti-nutrient. Several genes involved in the biosynthesis of PA biosynthesis (162) are identified, which needs to be suppressed through genome editing tools for reduced PA accumulation and maximum bioavailability of micronutrients (Figure 4). However, anti-nutrients compounds are crucial for various plant processes specifically biotic and abiotic stress tolerance, their knocking out will impair those processes as well and there will be a need for tradeoffs (53). Another aspect of biofortification that needs consideration is exploration of pathways and the role of different genes involved in adsorption of micronutrient from the soil, its translocation to the shoot, remobilization of the reserves to the reproductive part, and its bioavailability to the consumer (99). This could be achieved by using the association studies, i.e., genome wide association studies which are paving the way for fast track identification of candidate genes involved in various metabolic pathways (142).

As far as maize crop is concerned, three factors are of prime importance concerning biofortification, i.e., quality protein maize (QPM, having high lysine and tryptophan contents), pro-vitamin A, and zinc concentrations. The exploration of biosynthetic pathways and genes involved thereof will help to unravel the genetic complexity underlying these mechanisms. The intervention of advanced genomics techniques, i.e., genome-wide association studies and comparative transcriptomics studies for gene identification followed by modifications of key genes through new breeding techniques, i.e., RNAi, overexpression, and genome editing, will open new avenues for biofortification of crops (44). Another important aspect concerning biofortification of crops is the utilization of crop wild relatives as source material for biofortification. Different studies have highlighted that the nutritional value of CWRs is high as compared to cultivated crops; hence, these must be given due consideration for the transfer of desired genes using genetic engineering (Box 1) (65, 163).

Several transgenic biofortified crops have been developed and are available in the research laboratories. Most of them are not commercialized due to low consumer acceptability and regulatory issues. Further, biosafety assessment agencies and regulatory bodies for GM crops demand an equal amount of capital as used in their development which is a big hurdle in their commercialization. Furthermore, the consumer has concerns regarding antibiotic resistance due to bacterial markers genes present in GM crops. These issues could be tackled either through the development of marker-free transgenic plants or the use of genome editing tools for the development of transgene-free plants. GE crops have to pass through a looser regulatory process in some countries with the exception of the E.U. (127).

One major hurdle in the way of pulses biofortification is that genetic variation is yet to be explored for protein and starch composition and level of micro- and macronutrients. Therefore, there is a need to launch large-scale studies to understand the genetic diversity of important macro- and micronutrients in global pulses germplasm to effectively utilize it for pulses biofortification (4). The focus should be on increasing the bioavailability of micronutrients alongside increasing their concentration. For that purpose, concentration of promotor substances (which increase absorption of minerals) should be increased and that of anti-nutrients should be decreased. The promotor substances are vitamin A, C, D, and E, choline, and niacin, which increases the absorption of Se, Ca, P, Zn, Fe, methionine, and tryptophan (5).

Post-harvest management of crops is also in serious trouble in delivering safer foods to the consumer. Masses are unaware about dietary and nutritional significance of different parts of plants. For example, milled grain of most cereals is consumed around the globe. Although most of the essential elements, i.e., Se and S, are higher in germ but other essential elements, i.e., copper, zinc, and iron, are found in ample quantity in bran which is removed during the milling process and is not accessible to humans (164). Most micronutrients are found in the aleurone layers of the cereals which are removed during the milling process alongside bran. As a result, these are not available for consumption. This issue needs special attention and could be tackled in two ways. Whole-grain processing is the first option that will increase the nutritional value of products obtained afterward. The second option may be the engineering of biosynthetic pathways to alter the deposition of micronutrients from the aleurone layer to endosperm so that these may not be lost during milling and processing (165).

## Author Contributions

RS, SJ, and SA conceived the idea. RS, SJ, AN, SA, SKh, and ZA drafted the manuscript. SA and RS prepared illustrations. SKa, HMUA, RAG, and WZ provided the literature and technical assistance. RS, SJ, HMUA, SA, SKa, SKh, RAG, and WZ reviewed and improved the draft. All authors contributed to the article and approved the submitted version.

## Funding

This study is funded by Zhejiang Province Postdoctoral Research Project (ZJ2020141).

## Conflict of Interest

The authors declare that the research was conducted in the absence of any commercial or financial relationships that could be construed as a potential conflict of interest.

## Publisher's Note

All claims expressed in this article are solely those of the authors and do not necessarily represent those of their affiliated organizations, or those of the publisher, the editors and the reviewers. Any product that may be evaluated in this article, or claim that may be made by its manufacturer, is not guaranteed or endorsed by the publisher.
