# Supplementary material for: Biofortification of Cereals and Pulses Using New Breeding Techniques: Current and Future Perspectives
Source: Front Nutr. 2021 Oct 7;8:721728. doi: 10.3389/fnut.2021.721728 (PMC8528959; doi:10.3389/fnut.2021.721728)
Supplement: Supplementary file 1 [file Table_1.DOCX]

**Table S1. List of cereals and pulses varieties developed through conventional breeding other than HarvestPlus program for biofortification of different micronutrients for delivery of healthier diets to the masses.**

| **Crop** | **Micronutrient/ Anti-nutrient** | **Varieties Developed** | **Reference** |
| --- | --- | --- | --- |
| *Triticum aestivum* | Zinc | Zincol, NR42, 419 and 421, BHU1, 5,7 and 18, WB2, PBW1Zn, Akbar -2019 | ([Ortiz-Monasterio et al., 2007](#_ENREF_5)) |
|  | Iron | WB2 |  |
|  | Provitamin A | HI8627 |  |
| *Oryza sativa* | Zinc | Jalmegna, BRRI, dhan 62, 64,72 | ([Garg et al., 2018](#_ENREF_1)) |
|  | Iron | Jalmegne, improved line (IR68144-3B-2-2-3) | ([Majumder et al., 2019](#_ENREF_3)) |
|  | Phytic acid | Os-lpa-XS-110-1, Os-lpa-XS-110-2, Os-lpa-XQZ-1, | ([Perera et al., 2018](#_ENREF_6)) |
| *Zea mays* | Quality protein Maize | BR473, BR451, HQ31, HB proticta, HQ INTA 993, Obalampa, P70, CML140, FQH4567, CML194, Pusa Vivek QPM9, CML161, CML171, Pusa HQPM-5, Pusa HQPM-7, HKI1128Q, HM9, HM10Q, HM11Q, (HQPM-1-PV, HQPM-4-PV, HQPM-5-PV, HQPM-7-PV, HKI161-PV, HKI163-PV, HKI193-1-PV, and HKI193-2-PV, HQPM-5 | ([Muzhingi et al., 2017](#_ENREF_4); [Prasanna et al., 2020](#_ENREF_7)) |
|  | Vitamin A | CSIR-CRI honampa, Sammaz 39,Ife maizehyb 4, GV665A, GV662A, GV664A, GV665A GV662A, Abontem, MH39A, MH40A, ZS242A, RAHA02, Obatanpa, ZS261, BHQP542, Q623, Yanrui-1 | ([Muzhingi et al., 2017](#_ENREF_4); [Prasanna et al., 2020](#_ENREF_7)) |
|  | Zinc | BIO-MZN01, ICTA HB-15, ICTA B-15 |  |
| *Sorghum bicolor* | Iron | 12KNICSV 188, 12KNICSV 22, ICSH 14002, ICSR 14001 | ([Garg et al., 2018](#_ENREF_1); [Jha and Warkentin, 2020](#_ENREF_2)) |
| *Phaseolus vulgaris* | Zinc | MAC42, CAB2, RWV2887, 1129, 3317, 3006 and 33166, MAC44, RWR2154 | ([Garg et al., 2018](#_ENREF_1); [Jha and Warkentin, 2020](#_ENREF_2)) |
|  | Iron | PVA1438, HM21-7, CODMLB 32 and 001, RWR2245, Cuarentino. |  |
| *Lens culinaris* | Zinc | Idlib3, Idlib2, Alemaya, L4704, Shital, Simal, Sisir, Khajurah 2, ILL 7723, Barimasur 8, 4, 7 and 5, ILL 8006 X CDC Milestone | ([Garg et al., 2018](#_ENREF_1); [Jha and Warkentin, 2020](#_ENREF_2)) |
|  | Iron | Barimasur 8, 4, 7,5 and 6, Shital, Alemaya, Idlib2, ILL 7723, Shekhar, Khajurah 1, Pusa Vaibhav, ILL 8006 X CDC Milestone |  |
| *Common Bean* | Iron and Zinc | DOR364 X G19833, G14519 X G4825, G21242 X G21078 | ([Jha and Warkentin, 2020](#_ENREF_2)) |
| *Cicer arietinum* | Iron and Zinc | ICC 4958 X ICC 8261 | ([Jha and Warkentin, 2020](#_ENREF_2)) |
|  | Carotenoids | CDC Jade X CDC Frontier, Cory X CDC Jade, ICC4475 X CDC Jade | ([Jha and Warkentin, 2020](#_ENREF_2)) |
| *Pisum sativum* | Iron and Zinc | PI 648006 X PI 357292, Orb X CDC Striker, Carerra X CDC Striker, | ([Jha and Warkentin, 2020](#_ENREF_2)) |
|  | Selenium | Orb X CDC Striker, Carerra X CDC Striker | ([Jha and Warkentin, 2020](#_ENREF_2)) |

Garg, M., Sharma, N., Sharma, S., Kapoor, P., Kumar, A., Chunduri, V., and Arora, P., 2018. Biofortified crops generated by breeding, agronomy, and transgenic approaches are improving lives of millions of people around the world. Frontiers in nutrition 5**,** 12.

Jha, A.B., and Warkentin, T.D., 2020. Biofortification of pulse crops: Status and future perspectives. Plants 9**,** 73.

Majumder, S., Datta, K., and Datta, S.K., 2019. Rice Biofortification: High Iron, Zinc, and Vitamin-A to Fight against “Hidden Hunger”. Agronomy 9**,** 803.

Muzhingi, T., Palacios‐Rojas, N., Miranda, A., Cabrera, M.L., Yeum, K.J., and Tang, G., 2017. Genetic variation of carotenoids, vitamin E and phenolic compounds in Provitamin A biofortified maize. Journal of the Science of Food and Agriculture 97**,** 793-801.

Ortiz-Monasterio, J.I., Palacios-Rojas, N., Meng, E., Pixley, K., Trethowan, R., and Pena, R., 2007. Enhancing the mineral and vitamin content of wheat and maize through plant breeding. Journal of Cereal Science 46**,** 293-307.

Perera, I., Seneweera, S., and Hirotsu, N., 2018. Manipulating the phytic acid content of rice grain toward improving micronutrient bioavailability. Rice 11**,** 1-13.

Prasanna, B.M., Palacios-Rojas, N., Hossain, F., Muthusamy, V., Menkir, A., Dhliwayo, T., Ndhlela, T., San Vicente, F., Nair, S.K., and Vivek, B.S., 2020. Molecular breeding for nutritionally enriched maize: status and prospects. Frontiers in genetics 10**,** 1392.
